# Measuring catatonia motor behavior with objective instrumentation

**DOI:** 10.3389/fpsyt.2022.880747

**Published:** 2022-08-17

**Authors:** Sofie von Känel, Niluja Nadesalingam, Danai Alexaki, Daniel Baumann Gama, Alexandra Kyrou, Stéphanie Lefebvre, Sebastian Walther

**Affiliations:** ^1^Translational Research Center, University Hospital of Psychiatry, University of Bern, Bern, Switzerland; ^2^Graduate School for Health Sciences (GHS), University of Bern, Bern, Switzerland; ^3^Klinik Sonnenhalde, Basel, Switzerland

**Keywords:** actigraphy, schizophrenia, motor abnormalities, BFCRS, personalized medicine

## Abstract

**Objective:**

Catatonia is a neuropsychiatric syndrome, with important psychomotor features, associated with schizophrenia and other psychiatric disorders. The syndrome comprises multiple symptoms including abnormal motor control, behaviors, volition, and autonomic regulation. Catatonia assessment relies on clinical rating scales and clinicians familiar with the catatonia exam. However, objective instrumentation may aid the detection of catatonia. We aimed to investigate the relationship between movement parameters derived from actigraphy and expert ratings of catatonia symptoms measured by the Bush Francis Catatonia Rating Scale (BFCRS) and the Northoff Catatonia scale (NCS).

**Methods:**

Eighty-six acutely ill inpatients with schizophrenia spectrum disorders were assessed with the BFCRS, the NCS, and 24 h continuous actigraphy. Non-wear and sleep periods were removed from the actigraphy data prior to analysis. Associations between total catatonia scores, derived from both BFCRS and NCS, and actigraphy parameters as well as between single BFCRS items and actigraphy parameters were calculated using Spearman's rank correlation and non-parametric ANCOVAs (Quade's ANCOVAs), respectively.

**Results:**

Both higher BFCRS total scores (*r* = 0.369, *p* = 0.006) and NCS total scores (*r* = 0.384, *p* = 0.004) were associated with lower activity levels (AL). Higher scores on single BFCRS items such as *immobility/stupor* or *staring* were linked to lower AL (*immobility/stupor*: *F* = 17.388, *p* < 0.001, η^2^ = 0.175; *staring*: *F* = 7.849, *p* = 0.001, η^2^ = 0.162) and lower metabolic equivalents of task (MET).

**Conclusion:**

Specific catatonia symptoms such as *immobility/stupor* and *staring* can be measured with actigraphy. This may aid the detection, staging, and monitoring of catatonia in clinical settings.

## Introduction

Catatonia is a complex neuropsychiatric syndrome that involves psychomotor abnormalities. Symptoms include increased or decreased gross motor activity, abnormal motor control, behavior, volition, and autonomic regulation (1–3). First described in 1874 by Karl Kahlbaum, for decades catatonia was exclusively considered as a subtype of schizophrenia, despite conflicting evidence (4). The Diagnostic and Statistical Manual of Mental Disorders 5 (DSM-5) has revised the classification of catatonia and now enables diagnosing catatonia as an independent syndrome or as specifier to multiple conditions (5, 6). This was a very important classification change, since catatonia is not only observed in schizophrenia spectrum disorders but also in mood disorders, autism, dementia, intoxications, and general medical conditions (3, 5–9). Catatonia is quite frequent in samples of mixed acute psychiatric inpatients with a prevalence of 9% (10) and an incidence of 10.6 episodes per 100,000 person-years (11).

Catatonia can either present as an acute episode or persist in chronic forms (12). In case of an acute episode effective treatment is possible with lorazepam and electroconvulsive therapy (ECT) (8, 13, 14). In chronic catatonia treatment is more difficult due to persistent symptoms of unknown etiology and the lack of response to benzodiazepines (15, 16). Despite current treatment options, catatonia is still associated with increased mortality (mortality rate of 9%), especially if catatonia is not diagnosed correctly (11, 17, 18). Therefore, early diagnosis of catatonia is crucial in order to prevent morbidity and mortality. However, the diagnosis of catatonia remains challenging and clinicians are likely to neglect catatonia symptoms (19–21).

Varying definitions of the syndrome and insufficient training have rendered diagnosing catatonia difficult (1, 6, 21, 22). Indeed, more than 40 different symptoms have been described in the context of catatonia (1, 17, 22). In clinical practice catatonia is either diagnosed by referring to the DSM-5 diagnostic criteria or by applying clinical rating scales such as the Bush Francis Catatonia Rating Scale (BFCRS) (23) or the Northoff Catatonia Scale (NCS) (24). Depending on the diagnostic tool used, a different set of catatonia symptoms with distinct total scores as well as different diagnostic criteria and cut-off scores is included. Furthermore, symptom patterns of catatonia may fluctuate over time, resulting in periods characterized by either excitement or withdrawal in the same patient (6). In addition, not only can catatonia symptoms fluctuate as a result of the underlying mental disorder, but symptoms might also be influenced or induced by antipsychotic medication (25–27). Since catatonia assessments with either the DSM-5 criteria or clinical rating scales typically focus on a single time point, they might fall short detecting the syndrome. Undetected fluctuations or assessments at less optimal time points increase the risk of missing or underestimating the severity of catatonia (28). Finally, the clinical examination informs the clinical rating scale scoring or DSM checklists, but examination is subject to observation bias (29) and depends on the level of training (21, 28).

Instrumental measures have the potential to overcome diagnostic challenges of psychomotor abnormalities such as catatonia. Compared to clinical assessments, instrumental measures lack observer bias, require little training, and offer continuous long-term assessments. Furthermore, instrumental measures are highly reliable and sensitive, even in detecting subclinical motor abnormalities (28). Wrist actigraphy is a simple instrumental method to measure general physical activity across settings (28, 30). Moreover, wrist actigraphy enables the simultaneous measurement of different parameters reflecting physical activity (31). Multiple studies have demonstrated the use of wrist actigraphy in schizophrenia. For example, wrist actigraphy measures were associated with motor abnormalities such as antipsychotic induced akathisia or parkinsonism (32, 33) and psychopathological dimensions of schizophrenia (30, 31, 34). Furthermore, wrist actigraphy has been applied to study catatonia. In this context, wrist actigraphy indicated patients with the catatonic subtype of schizophrenia according to DSM-IV to have lower activity levels and longer periods of immobility compared to patients of the paranoid or disorganized subtype (35). In addition, increased catatonia severity, as measured by the BFCRS total score, was associated with lower activity levels (AL) (36).

Given that catatonia alters motor behavior in multiple ways, other actigraphy parameters such as the number of steps or metabolic parameters should be explored in relation to catatonia severity. Indeed, modern actigraphy technology offers more than one meaningful measure of physical activity, extending the possibilities of instrumental assessment of motor abnormalities in mental illness (27, 36, 37, 73). However, these associations have not been investigated so far. Likewise, the relationship between catatonia severity as measured by catatonia rating scales other than the BFCRS and actigraphy parameters remains unknown. Most research focuses on motor and volitional symptoms of catatonia (2), neglecting affective symptoms. Similarly, most clinical rating scales, including the BFCRS, focus on motor and behavioral symptoms of catatonia, again neglecting potential affective symptoms (38). Even though the NCS also includes affective symptoms, its association with measures of physical activity has not been investigated yet. Finally, catatonia is characterized by different and sometimes opposing symptom patterns (6, 9, 23), but currently it remains unclear which catatonia symptoms are measurable with actigraphy.

We aimed to investigate the association between the severity of catatonia indicated by the BFCRS as well as by the NCS and physical activity measured by actigraphy. We hypothesize that lower physical activity will be observed in cases of more severe catatonia. Furthermore, we aimed to examine the relationship between NCS motor, affective as well as behavioral subscores and physical activity measured through actigraphy. Here, we hypothesize that we will find the strongest association between the motor subscore and physical activity. Finally, we aimed to investigate whether the expected correlations between BFCRS total score and actigraphy parameters are specific to single items of the BFCRS. Again, we hypothesize that this might mainly be the case for core motor behavioral items.

## Materials and methods

### Participants

Eighty-six acutely ill patients with schizophrenia spectrum disorders according to the DSM-5 were recruited at the University Hospital of Psychiatry and Psychotherapy in Bern, Switzerland. All data included in this study are baseline data of an ongoing clinical trial named Overcoming Psychomotor Slowing in Psychosis (OCoPS-P, ClinicalTrials.gov Identifier: NCT03921450, funded by the Swiss National Science Foundation grant 182469). This trial is conducted according to the declaration of Helsinki and was approved by the local ethics committee (file 2018-02164). All participants provided written informed consent to participate in the whole clinical trial. Table 1 provides demographic and clinical characteristics of the patient sample. Exclusion criteria were active substance abuse or dependence other than nicotine, medical or neurological disorders affecting motor abilities, e.g., epilepsy, or past severe head trauma with temporary loss of consciousness.

**Table 1:** Demographic and clinical characteristics.

	**Patients (*n* = 86)**
**Demographics**	
Age (years)	35.1 ± 12.1
Gender (% female)	51.2
BMI (kg/m^2^)	25.9 ± 5.0
**Clinical characteristics**	
Medication (OLZ eq. in mg)	18.7 ± 2
Patients with typical antipsychotic medication	4 (4.7%)
Patients with atypical antipsychotic medication	70 (81.4%)
Patients with typical and atypical antipsychotic medication	10 (11.6%)
Patients with no antipsychotic medication	2 (2.3%)
BFCRS total score	4.7 ± 4.4
NCS total score	8.5 ± 5.3
NCS motor	1.9 ± 1.9
NCS affective	3.6 ± 2.3
NCS behavioral	2.9 ± 2.3

*BMI, Body mass index; kg/m^2^, kilograms per square meter; OLZ eq., olanzapine equivalents; BFCRS, Bush Francis catatonia rating scale; NCS, Northoff catatonia scale*.

All but 2 patients received antipsychotic treatment during data acquisition. Mean olanzapine equivalents (OLZ eq.) were calculated according to Gardner and Leucht (39–42).

### Assessments

#### Assessment of catatonia

Catatonia severity was assessed with the Bush Francis Catatonia Rating Scale (BFCRS) and the Northoff Catatonia scale (NCS). Both scales were rated consecutively by a single well-trained expert, i.e., advanced psychiatric residents (DA, DBG, AK). All raters were trained by the principal investigator to achieve optimal interrater reliability on the item levels (kappa > 0.8). The BFCRS consists of 23 items. Items 13 and 17–21 are rated as either absent or present with a score of 0 or 3, respectively. All other items are rated according to their severity on a scale from 0 to 3. On this scale 0 indicates absence, 1 occasional presence, 2 frequent presence, and 3 constant presence of the respective symptom. The BFCRS total score ranges from 0 to 69. The first 14 items of the BFCRS form the Bush Francis Catatonia Screening Instrument (BFCSI). The BFCSI is used to assess the presence or absence of catatonia. If two or more items on the BFCSI are rated as present irrespective of their severity, catatonia is diagnosed. The NCS consists of 40 items assessing catatonia symptoms in the motor (13 items), affective (12 items), and behavioral (15 items) domains. Catatonia is diagnosed if the NCS total score is >7 and a minimum of one symptom per domain (motor, affective, behavioral) irrespective of its severity is present. The items are rated on a scale from 0 to 2. On this scale 0 indicates absence, 1 the item is sometimes present, and 2 the item is always present. The total score of the NCS ranges from 0 to 80. Total subscores range from 0 to 26 for motor, 0 to 24 for affective, and 0 to 30 for behavioral catatonic symptoms.

#### Actigraphy

Actigraphy enables measuring multiple parameters related to physical activity. Activity level (AL), movement index (MI), and mean duration of uninterrupted immobility periods (MIP) are well-known actigraphy parameters based on activity counts derived from one axis of the acceleration signal (43). AL is the mean number of activity counts per hour. MI is the percentage of epochs with an activity count >0. MIP describes the amount and distribution of immobility epochs, i.e., epochs with an activity count of 0. Similar to AL, the number of steps indicates the total amount of physical activity, whereas similar to MI, the activity class (AC) indicates the total duration of active time. AC active refers to the percentage of time spent either walking or jogging and AC inactive refers to the percentage of waking time spent sedentary. However, contrary to MI, AC is based on different acceleration features and barometric air pressure (44). In addition, the actigraphy data provides information on metabolic parameters such as the intensity of physical activity based on metabolic equivalent of task (MET) classes and energy expenditure in general. MET is defined as the ratio between the amount of oxygen used by a person during physical activity in milliliters (mL) per kilogram of body mass and the reference value of 3.5 mL, which is equivalent to the assumed sedentary energy expenditure (45). MET <3 refers to light, whereas MET 3-6 refers to moderate, and MET >6 refers to vigorous physical activity (LPA, MPA, and VPA, respectively) (46). Resting energy expenditure (REE) refers to the energy used by a person when being completely sedentary. Active energy expenditure (AEE) refers to the energy used by a person when being physically active, i.e., everything requiring a MET >1. Total energy expenditure (TEE) is the sum of REE and AEE (47).

Patients wore a Move 4 actigraph (movisens GmbH; Karlsruhe, Germany) on the wrist of their non-dominant hand for 24 consecutive hours. The accelerometer produces voltage, whenever the device is moved in any direction (43). In order to determine the dominant hand, patients were assessed with the Edinburgh Handedness Inventory (48). Wearing the actigraph on the wrist of the non-dominant hand enables measuring general physical activity irrespective of manual work (43, 49).

### Data analysis

The actigraphy data were extracted using the movisens DataAnalyzer program with the modules Base, Physical Activity Metrics, and Energy Expenditure. The Base module enables the extraction of the step count and AC. The module Physical Activity Metrics allows for the extraction of the activity counts per minute needed for the calculation of AL, MI, and MIP. Finally, the Energy Expenditure module is used for the extraction of different MET classes and energy expenditure measures. All data were stored in 1 min epochs. Non-wear time (automatically flagged by the extraction software) during the 24 h data acquisition was removed prior to further analysis. Sixty-one patients (70.9%) wore the actigraph for more than 23 h. Non-wear time was mainly due to showering or bathing. All values obtained in minutes were then calculated as percentage of total wear time of the respective patient. In addition, sleep time was removed from the data for parameters specific to wake time [steps, AL, movement index (MI), mean duration of uninterrupted immobility periods (MIP), activity class (AC)]. Sleep time (including naps) was identified by considering sleep log information provided by the patients and visually inspecting the actograms for any inconsistencies. If the sleep periods visible in the actogram failed to match the sleep periods indicated by the participant in the sleep record, the sleep time was adjusted accordingly. Inactive periods during the day were not considered as sleep time unless information about a nap was provided by the participant. The data were then transferred to our own Excel® templates (31, 50) for further analysis.

Statistical analysis was conducted using IBM SPSS version 27. Apart from the following exceptions the data were complete for all participants: For one patient the single item rating of *immobility/stupor* and *staring* was not collected. For a second patient it was not possible to distinguish wake and sleep periods and no sleep record was available. Therefore, the parameters, AL, MI, MIP, steps, and AC could not be calculated. Finally, for a third patient AEE and therefore TEE could not be extracted correctly from the actigraphy data. Patients with missing data were excluded list wise from the respective analysis. Shapiro-Wilk tests showed that none of the data were normally distributed. We calculated Spearman's rank correlation controlling for age, OLZ eq., and body mass index (BMI) between actigraphy parameters and BFCRS as well as NCS total and subscores. Correlations were corrected for multiple comparisons using the false discovery rate (FDR). The parameters age, OLZ eq., and BMI were selected as covariates because they are known to influence physical activity in patients with severe mental illnesses (36). We further calculated non-parametric ANCOVAs (Quade's ANCOVAs) comparing actigraphy parameters between rating scores of severity (either 0, 1, 2, or 3) on all single items of the BFCRS, whilst controlling for age, OLZ eq., and BMI. In case of more than 2 groups/scores the Bonferroni method was used as a *post-hoc* test to correct for multiple comparisons. If <3 patients had the same rating on one item (see Table 2A for frequencies of BFCRS items), the comparison was considered to provide insufficient information and therefore the ratings of *n* <3 were not included in the *post-hoc* tests.

**Table 2:** Frequencies of catatonia signs.

**A. Frequencies of Bush Francis catatonia rating scale (BFCRS) signs**.								
**Response**	**0 (absent)**		**1 (occasional)**		**2 (frequent)**		**3 (constant)**	
	***N***	**%**	***N***	**%**	***N***	**%**	***N***	**%**
Excitement	80	93.0	6	7.0	0	0	0	0
Immobility/Stupor	69	81.2	16	18.8	0	0	0	0
Mutism	86	100.0	0	0	0	0	0	0
Staring	49	57.6	20	23.5	16	18.8	0	0
Posturing/Catalepsy	72	83.7	9	10.5	5	5.8	0	0
Grimacing	67	77.9	15	17.4	4	4.7	0	0
Echopraxia/Echolalia	77	89.5	6	7.0	2	2.3	1	1.2
Stereotypy	71	82.6	12	14.0	2	2.3	1	1.2
Mannerism	71	82.6	8	9.3	7	8.1	0	0
Verbigeration	78	90.7	7	8.1	1	1.2	0	0
Rigidity	45	52.3	23	26.7	18	20.9	0	0
Negativism	84	97.7	2	2.3	0	0	0	0
Waxy Flexibility	85	98.8	0	0	0	0	1	1.2
Withdrawal	85	98.8	1	1.2	0	0	0	0
Impulsivity	80	93.0	6	7.0	0	0	0	0
Automatic Obedience	78	90.7	5	5.8	3	3.5	0	0
Mitgehen	70	81.4	0	0	0	0	16	18.6
Gegenhalten	81	94.2	0	0	0	0	5	5.8
Ambitendency	71	82.6	0	0	0	0	15	17.4
Grasp Reflex	85	98.8	0	0	0	0	1	1.2
Perseveration	75	87.2	0	0	0	0	11	12.8
Combativeness	86	100.0	0	0	0	0	0	0
Autonomic Abnormality	86	100.0	0	0	0	0	0	0
**B. Frequencies of Northoff catatonia scale (NCS) signs**.								
**Response**	**0 (absent)**			**1 (occasional)**			**2 (frequent)**	
	***N***	**%**		***N***	**%**		***N***	**%**
**Motor domain**								
Mannerism	67	77.9		13	15.1		6	7.0
Stereotypy	71	82.6		12	14.0		3	3.5
Festination	78	90.7		7	8.1		1	1.2
Athetotic movements	82	95.3		4	4.7		0	0
Dyskinesia	81	94.2		5	5.8		0	0
Gegenhalten	81	94.2		4	4.7		1	1.2
Posing	80	93.0		6	7.0		0	0
Catalepsy	75	87.2		10	11.6		1	1.2
Waxy Flexibility	83	96.5		3	3.5		0	0
Rigidity	46	53.5		25	29.1		15	17.4
Muscular Hypotonia	83	96.5		3	3.5		0	0
Sudden change in muscle tone	83	96.5		3	3.5		0	0
Akinesia	75	87.2		9	10.5		2	2.3
**Affective domain**								
Compulsive Emotions	82	95.3		4	4.7		0	0
Emotional Lability	67	77.9		16	18.6		3	3.5
Impulsivity	80	93.0		6	7.0		0	0
Aggression	84	97.7		2	2.3		0	0
Excitement	64	74.4		20	23.3		2	2.3
Affect-/Emotion-related behavior	75	87.2		11	12.8		0	0
Flat Affect	16	18.6		34	39.5		36	41.9
Affect Latency	59	68.6		27	31.4		0	0
Anxiety	58	67.4		26	30.2		2	2.3
Ambivalence	61	70.9		21	24.4		4	4.7
Staring	51	59.3		28	32.6		7	8.1
Agitation	76	88.4		10	11.6		0	0
**Behavioral domain**								
Grimacing	66	76.7		18	20.9		2	2.3
Verbigeration	78	90.7		6	7.0		2	2.3
Perseveration	65	75.6		17	19.8		4	4.7
Increased, compulsive need for speech	73	84.9		12	14.0		1	1.2
Abnormal Language	62	72.1		20	23.3		4	4.7
Automatic Obedience	79	91.9		7	8.1		0	0
Echolalia/Echopraxia	79	91.9		5	5.8		2	2.3
Mitgehen	70	81.4		12	14.0		4	4.7
Compulsive behavior	85	98.8		1	1.2		0	0
Negativism	83	96.5		3	3.5		0	0
Autism/Withdrawal	36	41.9		27	31.4		23	26.7
Mutism	85	98.8		1	1.2		0	0
Stupor	83	96.5		3	3.5		0	0
Loss of decisiveness	54	62.8		27	31.4		5	5.8
Autonomic Abnormalities	86	100.0		0	0		0	0

## Results

Eighteen patients (15.5%) met criteria for catatonia according to DSM-5. According to the BFCSI 50 patients (58.1%) and according to the NCS 39 patients (45.3 %) of our sample could be diagnosed with catatonia syndrome. A total of 37 patients (43.0 %) could be diagnosed with catatonia syndrome by both the BFCSI and the NCS. Table 2 gives the frequencies of catatonia symptoms per scale. Most frequent items were *rigidity, staring*, and *grimacing* on the BFCRS and *flat affect* (affective domain), *autism/withdrawal* (behavioral domain), and *rigidity* (motor domain) on the NCS.

### Catatonia rating scales total scores

**Figure 1:**
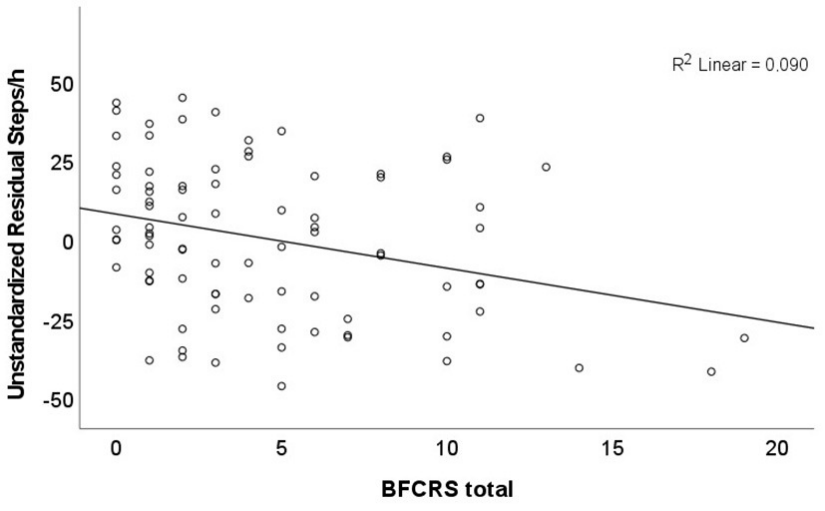
Association between Steps/h (steps per hour) and Bush Francis catatonia Rating Scale (BFCRS) total scores controlling for age, medication (olanzapine equivalents), and body mass index (BMI).

The BFCRS total score and the NCS total score correlated with each other (*r* = 0.815, *p* < 0.001). In addition, the BFCRS total score correlated with NCS subscores (motor domain: *r* = 0.705, *p* < 0.001; affective domain: *r* = 0.589, p < 0.001; behavioral domain: *r* = 0.647, *p* < 0.001). BFCRS and NCS total scores as well as the NCS behavioral subscore further correlated with AL, MI, steps (Figure 1 for BFCRS total score), AC, and multiple MET classes. In addition, the BFCRS total score further correlated with AEE and TEE. Whereas, the NCS affective subscore only correlated with AL, the NCS motor subscore failed to correlate with any of the physical or metabolic parameters (Table 3).

**Table 3:** Catatonia total and subscore correlations with actigraphy parameters controlling for age, medication (OLZ eq.), and body mass index (BMI).

	**AL/h**		**MI**		**MIP**		**Steps/h**		**AC: active**		**AC: inactive**	
	***p***	***r***	***p***	***r***	***p***	***r***	***p***	***r***	***p***	***r***	***p***	***r***
BFCRS total	0.006^**^	−0.369	0.041^*^	−0.234	0.894	−0.015	0.012^*^	−0.307	0.012^*^	−0.301	0.020^*^	0.275
NCS total	0.004^**^	−0.384	0.038^*^	−0.251	0.595	0.060	0.038^*^	−0.217	0.038^*^	−0.268	0.038^*^	0.248
NCS motor	0.464	−0.125	0.934	0.020	0.464	−0.097	0.464	−0.099	0.464	−0.116	0.464	0.105
NCS affective	0.012^*^	−0.375	0.054	−0.286	0.537	0.077	0.060	−0.248	0.060	−0.254	0.084	0.225
NCS behavioral	0.012^*^	−0.364	0.038^*^	−0.295	0.121	0.188	0.038^*^	−0.268	0.038^*^	−0.258	0.043^*^	0.247
	**REE**		**AEE**		**TEE**		**LPA (MET**<**3)**		**MPA (MET 3–6)**		**VPA (MET** >**6)**	
	***p***	***r***	***p***	***r***	***p***	***r***	***p***	***r***	***p***	***r***	***p***	***r***
BFCRS total	0.685	−0.054	0.012^*^	−0.293	0.033^*^	−0.247	0.008^**^	0.330	0.009^**^	−0.321	0.006^**^	−0.345
NCS total	0.578	0.070	0.123	−0.187	0.254	−0.139	0.038^*^	0.254	0.038^*^	−0.249	0.003^**^	−0.307
NCS motor	0.935	−0.009	0.464	−0.134	0.464	−0.114	0.464	0.101	0.464	−0.100	0.464	−0.190
NCS affective	0.581	0.061	0.327	−0.130	0.346	−0.119	0.150	0.188	0.150	−0.182	0.060	−0.250
NCS behavioral	0.286	0.124	0.218	−0.149	0.545	−0.068	0.038^*^	0.265	0.038^*^	−0.261	0.054	−0.231

* *Denotes significant correlations p < 0.05;*

** *denotes significant correlation p < 0.01*.

*OLZ eq., olanzapine equivalent; BFCRS, Bush Francis Catatonia Rating Scale; NCS, Northoff Catatonia Scale; AL/h, activity level per hour; MI, motor index; MIP, mean uninterrupted immobility periods; AC, activity class; REE, resting energy expenditure; AEE, active energy expenditure; TEE, total energy expenditure; LPA, light physical activity; MET, metabolic equivalent of task; MPA, moderate physical activity; VPA, vigorous physical activity*.

### Catatonia rating scales single item scores

**Figure 2:**
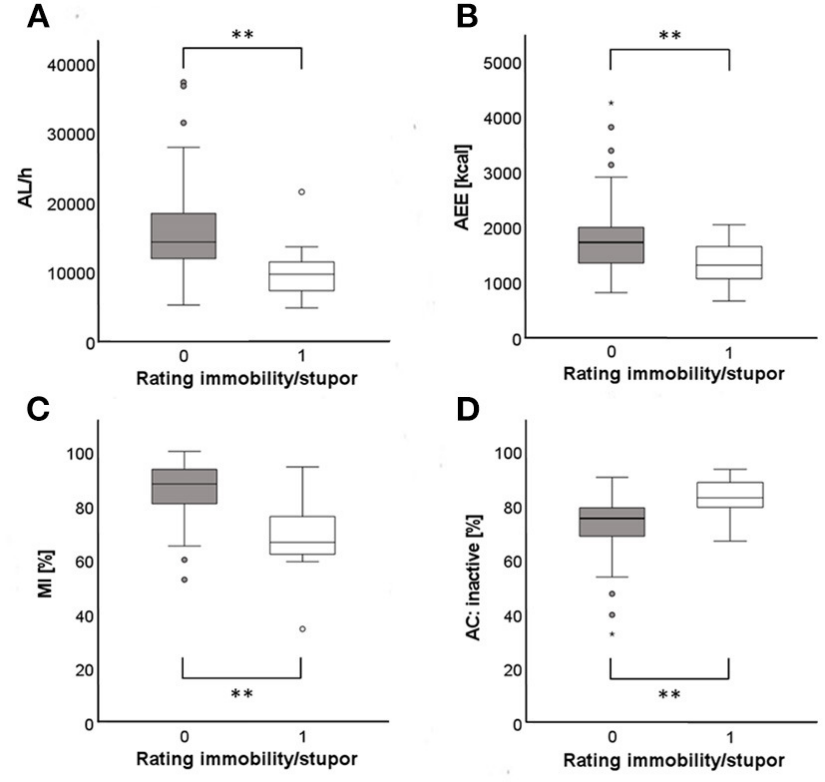
Group differences in AL/h **(A)**, AEE **(B)**, MI **(C)**, and AC: inactive **(D)** between absence (rating 0) and occasional presence (rating 1) of the item *immobility/stupor*. **Denotes significant correlation *p* < 0.01. AL/h, activity level per hour; AEE, active energy expenditure; MI, motor index; AC, activity class.

Results of the relationship between single items of the BFCRS and actigraphy parameters are displayed in Table 4. The items *immobility/stupor* and *staring* showed the most impressive relationship between their different severity ratings and actigraphy parameters. Comparison of absence (rating 0) and occasional presence (rating 1) of *immobility/stupor* showed a significant difference in all actigraphy parameters, except for REE. Examples for group differences in AL, AEE, MI, and AC are displayed in Figure 2. For different ratings of the item *staring* all actigraphy parameters, except MIP, REE, and TEE differed between groups. A *post-hoc* test revealed that for most actigraphy parameters significant differences in the rating of the item *staring* are present between absent and frequent staring but not between absent and occasional or occasional and frequent staring (AL: *p* = 0.001; MI: *p* = 0.011; steps: *p* = 0.025; MPA: *p* = 0.005; VPA: *p* = 0.027; AEE: *p* = 0.031). However, for LPA a *post-hoc* tests revealed that significant group differences are not only present between absent and frequent staring (*p* = 0.005) but also between occasional and frequent staring (*p* = 0.045). Even though there was no significant difference between the groups for *immobility/stupor* and *staring* when analyzing REE, we could detect a significant group difference for the single items *stereotypy* (F = 10.994, *p* = 0.001, η^2^ = 0.120 when only comparing absence and occasional presence), *rigidity* (no longer significant after *post-hoc* test), and *Mitgehen*. The latter also showed significant group differences for TEE. Finally, we detected significant group differences between absence and presence of *ambitendency* for all MET classes.

**Table 4:** Relationship between single BFCRS item ratings and physical or metabolic parameters controlling for age, medication (OLZ eq.), and body mass index (BMI).

	**AL/h**			**MI**			**MIP**			**Steps/h**		
	***F***	***p***	***η^2^***	***F***	***p***	***η^2^***	***F***	***p***	***η^2^***	***F***	***p***	***η^2^***
Excitement	0.138	0.711	0.002	1.386	0.242	0.016	2.170	0.145	0.025	2.342	0.130	0.027
Immobility/Stupor	17.388	<0.001^**^	0.175	18.699	<0.001^**^	0.186	4.007	0.049^*^	0.047	18.803	<0.001^**^	0.187
Staring	7.849	0.001^**^	0.162	5.403	0.006^**^	0.118	1.453	0.240	0.035	3.671	0.030^*^	0.083
Posturing/Catalepsy	0.962	0.386	0.023	0.575	0.565	0.014	0.748	0.477	0.018	0.168	0.845	0.004
Grimacing	2.180	0.120	0.050	0.663	0.518	0.016	0.971	0.383	0.023	1.805	0.171	0.042
Echopraxia/Echolalia	1.487	0.224	0.052	1.920	0.133	0.066	2.061	0.112	0.071	2.022	0.117	0.070
Stereotypy	1.661	0.182	0.058	0.319	0.812	0.012	0.143	0.934	0.005	0.942	0.424	0.034
Mannerism	0.033	0.967	0.001	0.710	0.495	0.017	0.864	0.425	0.021	0.409	0.665	0.010
Verbigeration	0.002	0.962	0.000	0.067	0.796	0.001	0.037	0.847	0.000	0.544	0.463	0.007
Rigidity	0.108	0.898	0.003	0.263	0.770	0.006	1.164	0.317	0.028	0.292	0.747	0.007
Impulsivity	0.039	0.845	0.000	0.081	0.776	0.001	0.669	0.416	0.008	0.032	0.858	0.000
Automatic Obedience	2.660	0.076	0.061	3.062	0.052	0.070	1.564	0.216	0.037	2.249	0.112	0.052
Mitgehen	1.025	0.314	0.012	1.539	0.218	0.018	0.008	0.929	0.000	1.281	0.261	0.015
Gegenhalten	0.482	0.489	0.006	0.521	0.472	0.006	0.532	0.468	0.006	1.452	0.232	0.017
Ambitendency	0.679	0.412	0.008	0.165	0.685	0.002	0.558	0.457	0.007	2.545	0.114	0.030
Perseveration	1.385	0.243	0.016	0.678	0.413	0.008	0.051	0.822	0.001	2.781	0.099	0.032
	**AC: active**			**AC: inactive**			**REE**			**AEE**		
	***F***	***p***	*η^2^*	***F***	***p***	*η^2^*	***F***	***p***	*η^2^*	***F***	***p***	*η^2^*
Excitement	1.144	0.288	0.014	0.967	0.328	0.012	0.488	0.487	0.006	0.471	0.495	0.006
Immobility/Stupor	13.400	<0.001^**^	0.140	14.327	<0.001^**^	0.149	2.433	0.123	0.028	7.454	0.008^**^	0.083
Staring	3.806	0.026^*^	0.086	4.022	0.022^*^	0.090	0.142	0.868	0.003	3.510	0.035^*^	0.080
Posturing/Catalepsy	0.155	0.857	0.004	0.181	0.835	0.004	0.068	0.934	0.002	0.998	0.373	0.024
Grimacing	1.656	0.197	0.039	1.874	0.160	0.044	2.015	0.140	0.046	0.170	0.844	0.004
Echopraxia/Echolalia	1.054	0.373	0.038	1.198	0.316	0.042	1.554	0.207	0.054	0.571	0.636	0.021
Stereotypy	0.970	0.411	0.035	0.983	0.405	0.035	4.393	0.006^*^	0.138	1.348	0.265	0.048
Mannerism	0.032	0.968	0.001	0.055	0.947	0.001	1.639	0.200	0.038	0.259	0.773	0.006
Verbigeration	0.094	0.760	0.001	0.157	0.693	0.002	0.172	0.842	0.004	1.138	0.326	0.027
Rigidity	0.106	0.899	0.003	0.265	0.768	0.006	3.154	0.048^*^	0.071	1.136	0.326	0.027
Impulsivity	0.002	0.968	0.000	0.000	0.997	0.000	1.995	0.161	0.023	0.240	0.626	0.003
Automatic Obedience	1.369	0.260	0.032	1.430	0.245	0.034	0.711	0.494	0.017	1.485	0.233	0.035
Mitgehen	0.912	0.342	0.011	0.969	0.328	0.012	5.985	0.017^*^	0.067	2.485	0.119	0.029
Gegenhalten	2.817	0.097	0.033	0.896	0.347	0.011	1.666	0.200	0.019	0.089	0.766	0.001
Ambitendency	2.636	0.108	0.031	1.407	0.239	0.017	0.358	0.551	0.004	0.843	0.361	0.010
Perseveration	3.964	0.050	0.046	2.145	0.147	0.025	0.048	0.828	0.001	0.144	0.706	0.002
	**TEE**			**LPA (MET**<**3)**			**MPA (MET 3–6)**			**VPA (MET** >**6)**		
	***F***	***p***	*η^2^*	***F***	***p***	*η* ^2^	***F***	***p***	*η* ^2^	***F***	***p***	*η* ^2^
Excitement	0.099	0.754	0.001	0.094	0.760	0.001	0.170	0.681	0.002	0.064	0.802	0.001
Immobility/Stupor	6.520	0.013^*^	0.074	13.724	<0.001^**^	0.142	13.362	<0.001^**^	0.139	4.310	0.041^*^	0.049
Staring	1.976	0.145	0.047	5.434	0.006^**^	0.117	5.320	0.007^**^	0.115	3.785	0.027^*^	0.085
Posturing/Catalepsy	0.576	0.564	0.014	1.022	0.364	0.024	0.869	0.423	0.021	2.625	0.078	0.059
Grimacing	0.322	0.726	0.008	0.671	0.514	0.016	0.812	0.447	0.019	0.603	0.550	0.014
Echopraxia/Echolalia	0.732	0.536	0.026	1.404	0.248	0.049	1.398	0.249	0.049	0.947	0.422	0.033
Stereotypy	1.300	0.280	0.046	1.425	0.241	0.050	1.312	0.276	0.046	2.220	0.092	0.075
Mannerism	0.327	0.722	0.008	0.174	0.841	0.004	0.177	0.838	0.004	0.666	0.517	0.016
Verbigeration	0.927	0.400	0.022	0.886	0.416	0.021	0.861	0.427	0.020	1.024	0.364	0.024
Rigidity	1.203	0.305	0.029	0.362	0.697	0.009	0.367	0.694	0.009	0.545	0.582	0.013
Impulsivity	0.826	0.366	0.010	0.206	0.651	0.002	0.285	0.595	0.003	0.005	0.944	0.000
Automatic Obedience	1.326	0.271	0.031	1.977	0.145	0.045	2.026	0.138	0.047	0.346	0.709	0.008
Mitgehen	4.261	0.042^*^	0.049	2.397	0.125	0.028	2.307	0.133	0.027	2.604	0.110	0.030
Gegenhalten	0.015	0.904	0.000	1.389	0.242	0.016	1.202	0.276	0.014	0.319	0.574	0.004
Ambitendency	0.197	0.659	0.002	4.648	0.034^*^	0.052	4.341	0.040^*^	0.049	5.056	0.027^*^	0.057
Perseveration	0.051	0.821	0.001	1.595	0.210	0.019	1.494	0.225	0.017	2.080	0.153	0.024

* *Denotes significant correlations p < 0.05;*

** *denotes significant correlation p < 0.01*.

*BFCRS, Bush Francis Catatonia Rating Scale OLZ eq., olanzapine equivalent; AL/h, activity level per hour; MI, motor index; MIP: mean uninterrupted immobility periods; AC, activity class; REE, resting energy expenditure; AEE, active energy expenditure; TEE, total energy expenditure; LPA, light physical activity; MET, metabolic equivalent of task; MPA, moderate physical activity; VPA, vigorous physical activity*.

## Discussion

Catatonia remains a complex syndrome, challenging clinicians during detection and monitoring. Instrumental motor assessments such as actigraphy bear the potential to reduce complexity and add confidence in the clinical setting. This study aimed to test the correspondence of multiple actigraphy measures with two catatonia rating scales. In line with our hypothesis catatonia measured by both the BFCRS and the NCS is associated with instrumental measures of physical activity. Likewise, the NCS behavioral subscore correlated with different physical and metabolic parameters. Contrary to our hypothesis, the NCS motor subscore failed to correlate with any of the actigraphy measures, whereas the NCS affective subscore only correlated with the AL. On a single item level, actigraphy parameters differed most impressively between severity ratings of BFCRS items *immobility/stupor* and *staring*, indicating superior external validity of these items.

Correlations between catatonia severity and the amount of physical activity corroborate the only other study showing an association between a higher BFCRS total score and a lower AL in a different sample with a different actigraphy instrument than the current one (36). Additionally, our findings extend previous knowledge by considering novel parameters reflecting physical activity and examining both the BFCRS and the NCS. Differences in the definition and selection of catatonia symptoms are most likely the reasons why the BFCRS but not the NCS total score correlated with energy expenditure measures. Compared to the BFCRS items some of the NCS items are defined differently. For example, in the BCRS *immobility/stupor* is defined as extreme hypoactivity starting with a rating of 1 when patients sit abnormally still, whereas in the NCS *stupor* is defined as akinesia lasting for at least 30 min. Furthermore, compared to the BFCRS the NCS comprises more non-motor catatonia symptoms, e.g., affective symptoms (24). Indeed, not all catatonia symptom domains appear to be similarly measurable with actigraphy. Whereas, the NCS behavioral subscore correlated with parameters indicating the amount, duration, and intensity of physical activity, the NCS affective subscore only correlated with AL. Moreover, the NCS motor subscore failed to correlate with any of the actigraphy measures. It is important to note that some items of the NCS motor domain cover motor behaviors that are unlikely to alter total motor activity, e.g., *muscular hypotonus, sudden changes of muscular tone, athetotic movements* or *festination*. Thus, the selection of the NCS motor domain items might render associations with actigraphy measures difficult. Likewise, a study investigating the relationship between actigraphy parameters (AL, MI, MIP) and Positive and Negative Syndrome Scale (PANSS) scores did not detect any significant correlations between the PANSS motor retardation items and physical activity. Similarly, PANSS motor retardation items cover multiple issues including the speed of thoughts and speech and therefore extend core motor behavior (31). In line with our hypothesis the motor symptom *immobility/stupor* showed group differences in most actigraphy parameters, including the MIP. In fact, increased MIP, i.e., longer duration of immobility, has been proposed as a specific feature of catatonia in categorical comparisons (35). However, no correlation was found for MIP and subscores or total scores of catatonia rating scales, arguing for the analysis of single rating scale items or behaviors when investigating the pathophysiology of catatonia (2, 28). Regarding the single item *staring* the fact that group differences were only observed between absent and frequent *staring* implies that this symptom needs to be present at a more severe level in order to be measurable with actigraphy. Nonetheless, both *immobility/stupor* and *staring* were among the most frequent catatonia symptoms not only in our sample but also in a larger mixed clinical sample (51). Therefore, both single items should be taken into account in future investigations of the relationship between catatonia and physical activity.

Lower physical activity in schizophrenia patients with catatonia has been attributed to decreased motor output resulting from functional and structural abnormalities in the motor circuitry (6, 38, 50–59). Findings indicate that key parts of the motor circuit are also critically altered in catatonia, rendering the system less flexible for environmental adaptation. Furthermore, sedentary behavior might induce deterioration of the already decreased motor output due to lack of training (36). Indeed, a beneficial effect of exercise in chronic catatonia has been proposed anecdotally (15). Furthermore, motor abnormalities in catatonia comprise both hypokinetic and hyperkinetic movements, suggesting a dysfunction in multiple motor circuits (6). Moreover, as actigraphy provides a direct measure of motor behavior, it may critically inform studies on the neural correlates of motor abnormalities as demonstrated previously by our group and others (37, 50, 52, 53, 56, 60, 61). However, most research, including neuroimaging studies, focuses on motor and volitional symptoms of catatonia (2). Interestingly, studies relying on motor/behavioral rating scales such as the BFCRS show aberrant functioning of a core motor circuit including cortical and subcortical areas mediated by dopamine. In contrast, studies relying on the NCS show aberrant functioning in higher-order frontoparietal networks mediated by gamma-aminobutyric acid (GABA) and glutamate (38). This again underlines the importance of analyzing the heterogeneous catatonia symptoms separately in order to elucidate the underlying pathophysiology of catatonia behaviors.

Despite rich literature on actigraphy in the assessment of sleep or sleep-wake patterns and circadian rhythms (62), data on actigraphy measures in motor and psychomotor disorders is still limited. However, measuring catatonia symptoms with actigraphy might aid the detection and staging of catatonia in clinical settings. In schizophrenia, associations between physical activity and negative syndrome (30, 31, 63), dosage of antipsychotic medication (64), and the development of motor activity over the course of schizophrenia (65) have been investigated. Besides catatonia, actigraphy has been implemented in the investigation of other motor abnormalities such as (antipsychotic-induced) akathisia (32, 66), parkinsonism (36, 66), dystonia (66), and restless legs syndrome (67). Furthermore, psychomotor behaviors in other mental disorders such as major depressive disorder (MDD) have been investigated using actigraphy (68–70).

The strengths of the study include objective assessments of physical activity represented by multiple parameters and clinical assessments of catatonia with different rating scales. Furthermore, all results were controlled for medication (OLZ eq.), age, and BMI, all potential confounders of physical activity (36, 64). This study is limited by the moderate sample size, cross-sectional assessments, focus on schizophrenia spectrum disorders, and inclusion of a small number of severe catatonia cases. Specifically, since our data is derived from a randomized controlled trial on psychomotor slowing, all patients included consented to participating in a clinical trial, which is less likely than participation in our previous observational studies in catatonia (35, 36, 55–57). Therefore, generalization of our findings to all catatonia patients might be limited due to potential selection bias. Even though a large proportion of our patients already displayed reduced movement due to psychomotor slowing, associations between catatonia and physical activity were still present. This suggests that in samples of patients without psychomotor slowing associations between catatonia and physical activity might become even more prominent. On the other hand, the generalization of our findings might be also limited due to our sample comprising mostly patients with psychomotor slowing. Moreover, the observed associations between catatonia symptoms and physical activity mainly apply to hypokinetic forms of catatonia and require further testing in hyperkinetic catatonia. Finally, our data did not allow for the distinction between genuine and drug-related catatonia, and we were unable to investigate potential confounding effects on catatonia symptoms caused by other drug-related motor abnormalities such as parkinsonism. Future studies testing the application of actigraphy for screening and staging of catatonia should include catatonia patients with multiple underlying conditions and investigate different forms of catatonia. In addition, long-term assessments covering multiple days are needed to address the issue of fluctuating symptoms in catatonia (6). Finally, disease mechanisms of reduced physical activity in catatonia might be elucidated using brain stimulation and other interventional studies, e.g., combining physical exercise and neuroimaging (71, 72).

## Conclusion

The severity of hypokinetic catatonia is associated with a decreased amount, duration, and intensity of physical activity as well as a decreased energy expenditure. Multiple actigraphy parameters offer important insights. Furthermore, our results suggest that actigraphy may capture specific symptoms better than composite scores of multiple items. Whereas, both BFCRS and NCS total scores were linked to physical activity, the optimal actigraphy parameters to monitor catatonia are yet to be determined. In the future, measuring catatonia symptoms with actigraphy may aid the detection and staging of catatonia in clinical settings as well as the monitoring of treatment effects in clinical trials.

## Data availability statement

The datasets presented in this article are not readily available because not all participants of the studies consented to data sharing. As these are personal medical data, sharing is only possible upon informed consent. Requests to access the datasets should be directed to SW, sebastian.walther@upd.unibe.ch.

## Ethics statement

The studies involving human participants were reviewed and approved by Kantonale Ethikkommission Bern (KEK). The patients/participants provided their written informed consent to participate in this study.

## Author contributions

SK analyzed the data and wrote the first draft of the manuscript. NN recruited participants and conducted assessments. DA, DB, and AK conducted clinical assessments. SL supervised assessments. SW designed the study, obtained funding, wrote the protocol, supervised assessments, analyzed, and edited the manuscript. All authors discussed the findings and edited the manuscript.

## Funding

This study received funding from the Swiss National Science Foundation, grant #182469 to SW. The funder had no access to the data or influence on the decision to publish.

## Conflict of interest

SW received honoraria from Janssen, Lundbeck, Mepha, Neurolite, and Sunovion, which were unrelated to this work. The remaining authors declare that the research was conducted in the absence of any commercial or financial relationships that could be construed as a potential conflict of interest.

## Publisher's note

All claims expressed in this article are solely those of the authors and do not necessarily represent those of their affiliated organizations, or those of the publisher, the editors and the reviewers. Any product that may be evaluated in this article, or claim that may be made by its manufacturer, is not guaranteed or endorsed by the publisher.
